# Supplementary material for: Biophysical mechanisms of default mode network function and dysfunction
Source: bioRxiv. 2026 Feb 16:2025.04.16.649208. Preprint. [Version 2] doi: 10.1101/2025.04.16.649208 (PMC12934715; doi:10.1101/2025.04.16.649208)
Supplement: Supplement 1 [file NIHPP2025.04.16.649208v2-supplement-1.pdf]

bioRxiv preprint doi: https://doi.org/10.1101/2025.04.16.649208; this version posted February 16, 2026. The copyright holder for this preprint (which was not certified by peer review) is the author/funder, who has granted bioRxiv a license to display the preprint in perpetuity. It is made available under aCC-BY 4.0 International license.

Supplementary Materials

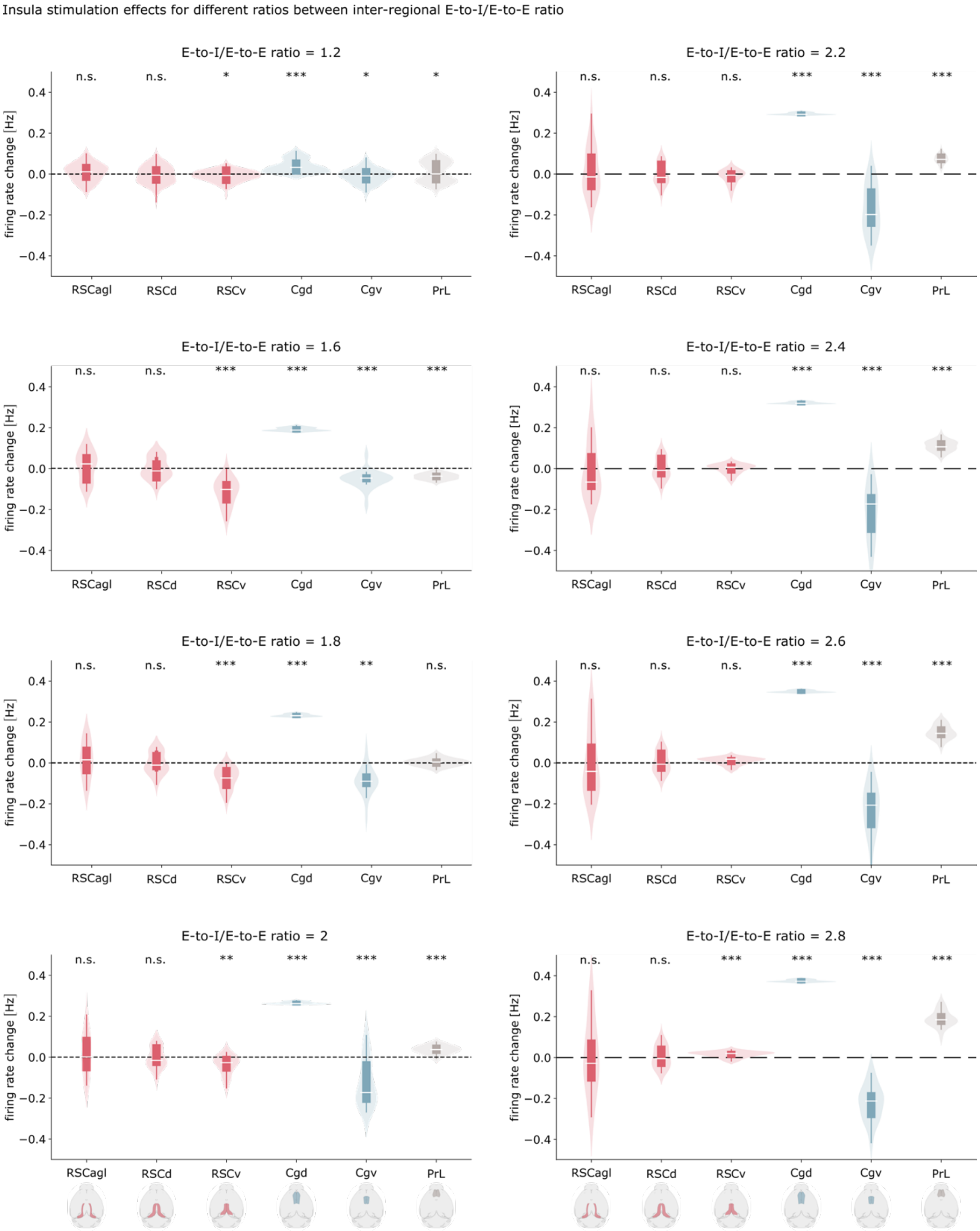

Figure S1. Insula stimulation produces coordinated DMN suppression across a range of ratios between E-to-E and E-to-I inter-regional projection weights. The RSC is found to be suppressed RSCv across ratios from 1.2 to 2.2, the Cgv across ratios from 1.2 to 2.8, and the PrL across ratios from 1.2 to 1.8. The results show that DMN suppression is robustly obtained ratios provided stronger E-to-I than E-to-E weights.

bioRxiv preprint doi: https://doi.org/10.1101/2025.04.16.649208; this version posted February 16, 2026. The copyright holder for this preprint (which was not certified by peer review) is the author/funder, who has granted bioRxiv a license to display the preprint in perpetuity. It is made available under aCC-BY 4.0 International license.

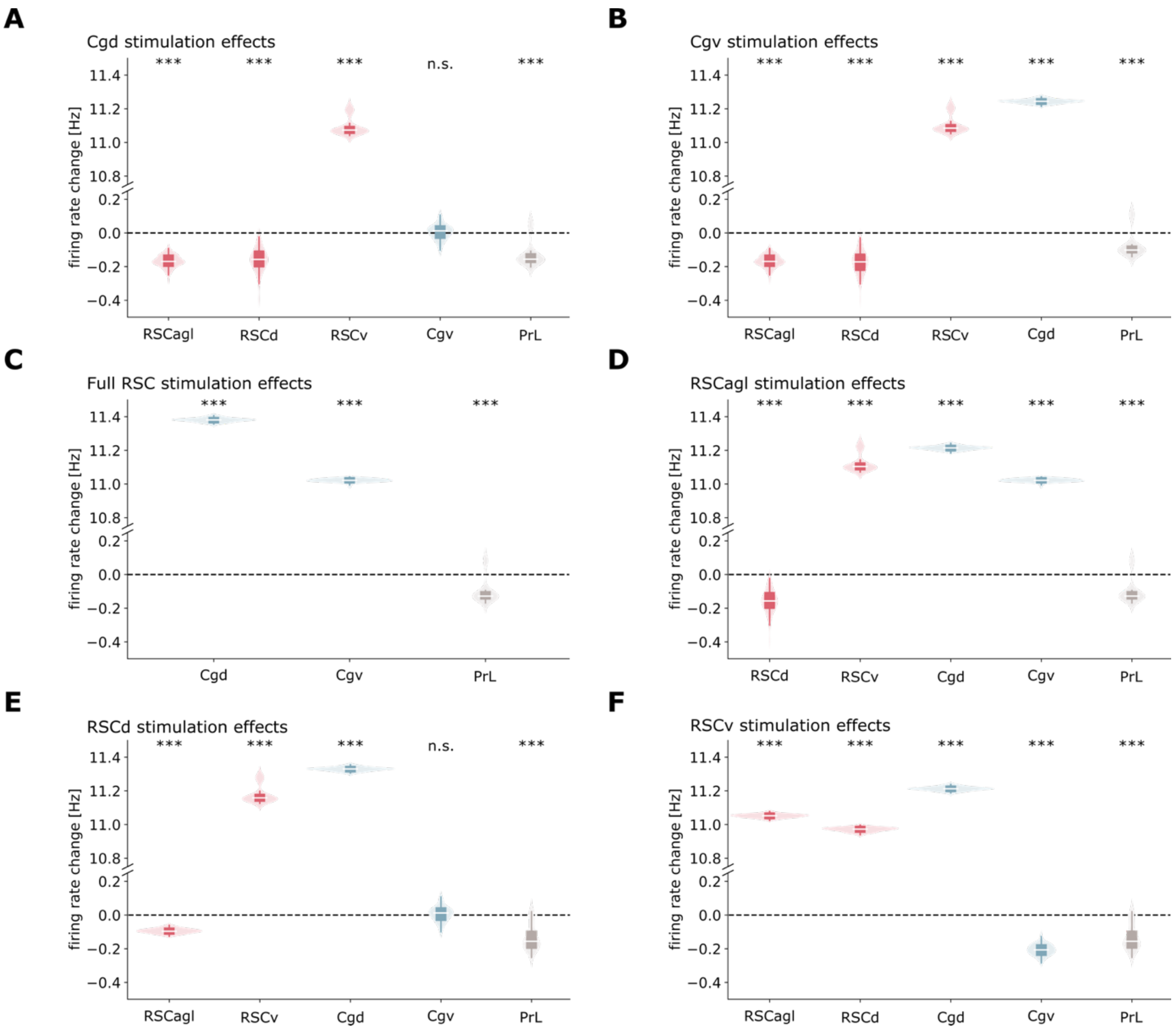

Figure S2. Effects of RSC and Cg subdivision stimulation on DMN activity. (A-B) Stimulation of the Cgd **(A)** and Cgd **(B)** both enhance RSCv dynamics while suppressing other RSC subdivisions as well as the PrL. **(C-F)** Stimulation of the full RSC **(C)** as well as of its RSCagl **(D)**, RSCd **(E)**, and RSCv **(F)** subdivisions separately all enhance the Cgd and suppress the PrL. Effects on the Cgv are subdivision-specific, with net enhancement from full RSC stimulation. The results show the Cg and RSC, when stimulated, mutually enhance each other, unlike the insula when stimulated (Figure 2).

bioRxiv preprint doi: https://doi.org/10.1101/2025.04.16.649208; this version posted February 16, 2026. The copyright holder for this preprint (which was not certified by peer review) is the author/funder, who has granted bioRxiv a license to display the preprint in perpetuity. It is made available under aCC-BY 4.0 International license.

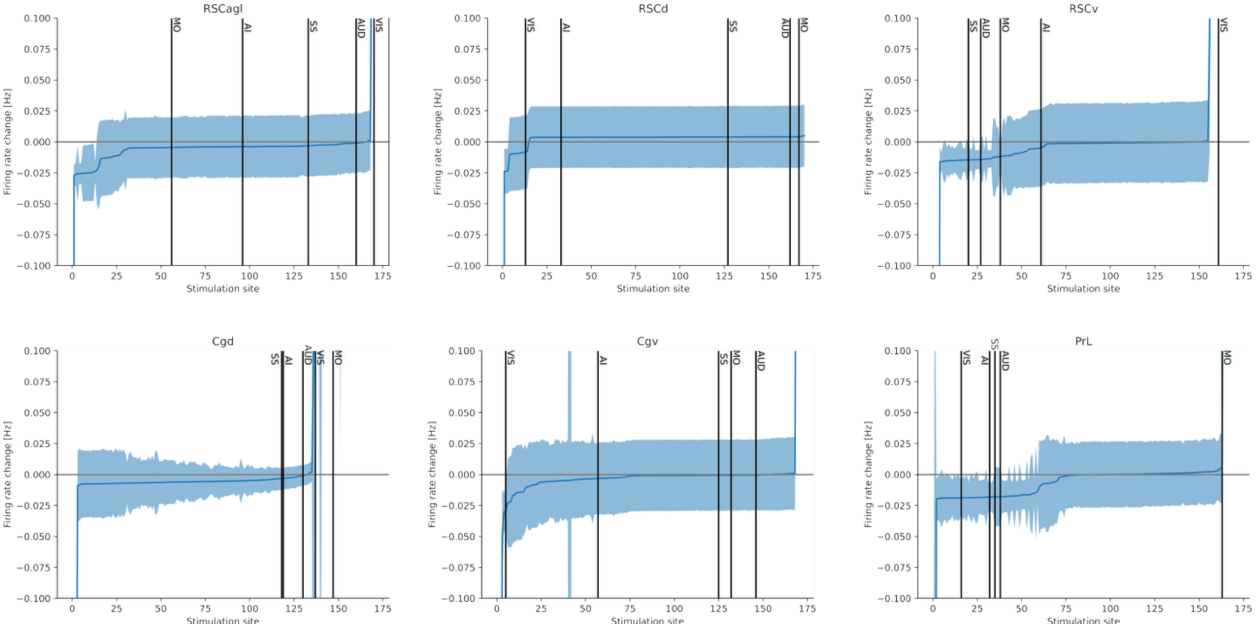

Figure S3. Effects of stimulation to each brain region on DMN activity. The simulated firing rate change in each brain region is shown as a function of the stimulation site. Different panels show firing rate change in different DMN subdivisions. Stimulation sites spanned all regions of the right hemisphere and are ranked in increasing order (from most negative to most positive) of firing rate change in each DMN subdivision and each panel. Black vertical lines indicate stimulation to the auditor (AUD), somatosensory (SS), visual (VIS), and motor (MO) cortex.

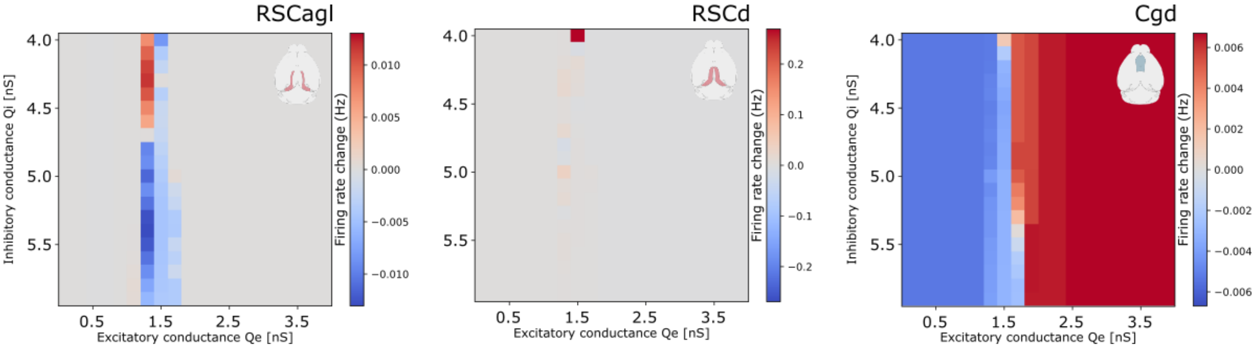

Figure S4. Parameter exploration of local E-I balance impacts on agranular and dorsal DMN subdivision suppression. Heatmaps of simulated subregional response to insula stimulation in the RSCagl (left), RSCd (middle), and Cg (right) as a function of E and I synaptic conductance in the RSC. The results show the region-specific and distributed effects of E-I imbalance in the RSC.

bioRxiv preprint doi: https://doi.org/10.1101/2025.04.16.649208; this version posted February 16, 2026. The copyright holder for this preprint (which was not certified by peer review) is the author/funder, who has granted bioRxiv a license to display the preprint in perpetuity. It is made available under aCC-BY 4.0 International license.

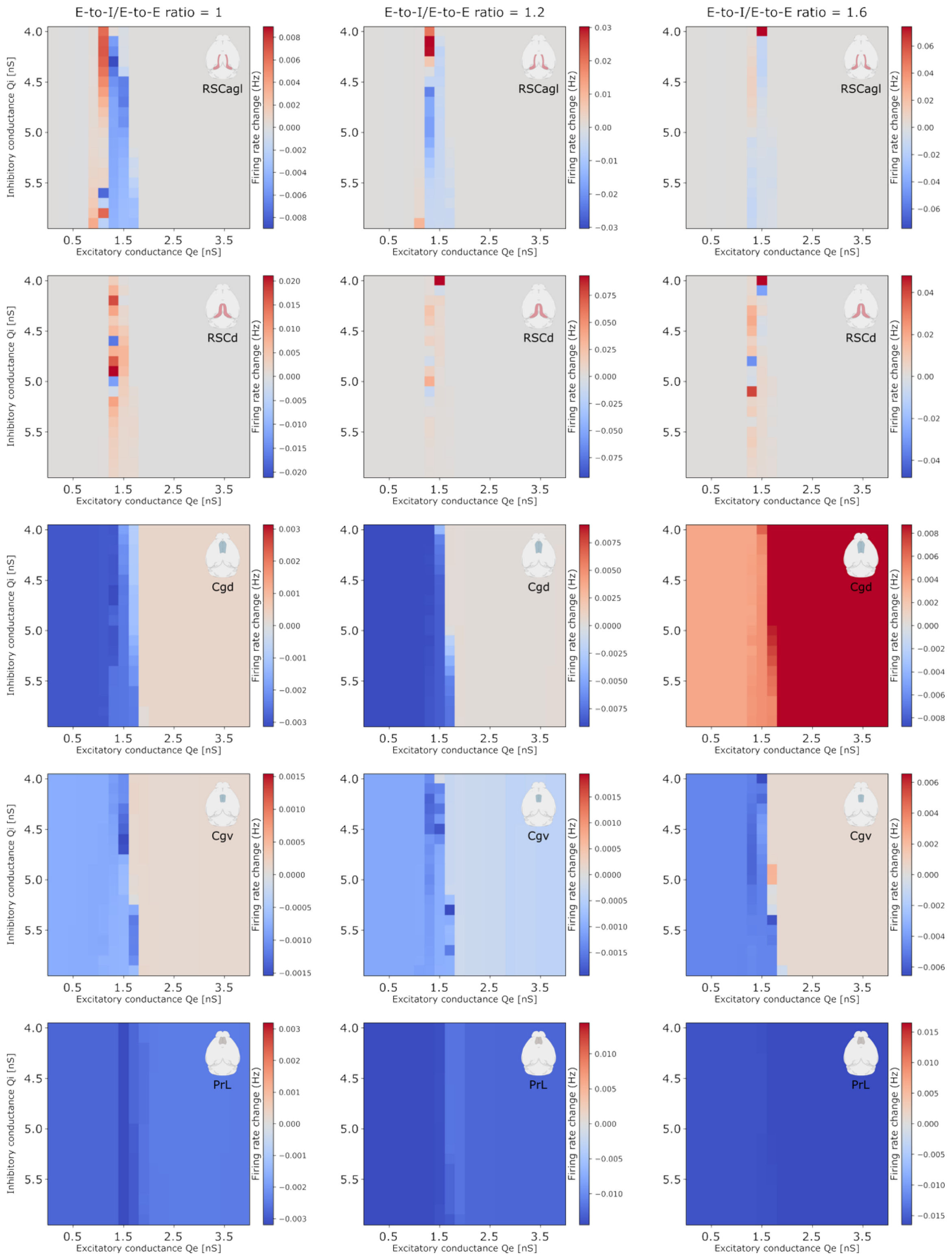

Figure S5. Effect of E-to-I/E-to-E ratio on DMN-wide suppression. Heatmaps of simulated responses in all DMN subregions except the RSCv, as a function of E and I synaptic conductance Q_E and Q_I in the RSC for a varying E-to-I/E-to-E ratio and zero spike-frequency adaptation.

bioRxiv preprint doi: https://doi.org/10.1101/2025.04.16.649208; this version posted February 16, 2026. The copyright holder for this preprint (which was not certified by peer review) is the author/funder, who has granted bioRxiv a license to display the preprint in perpetuity. It is made available under aCC-BY 4.0 International license.

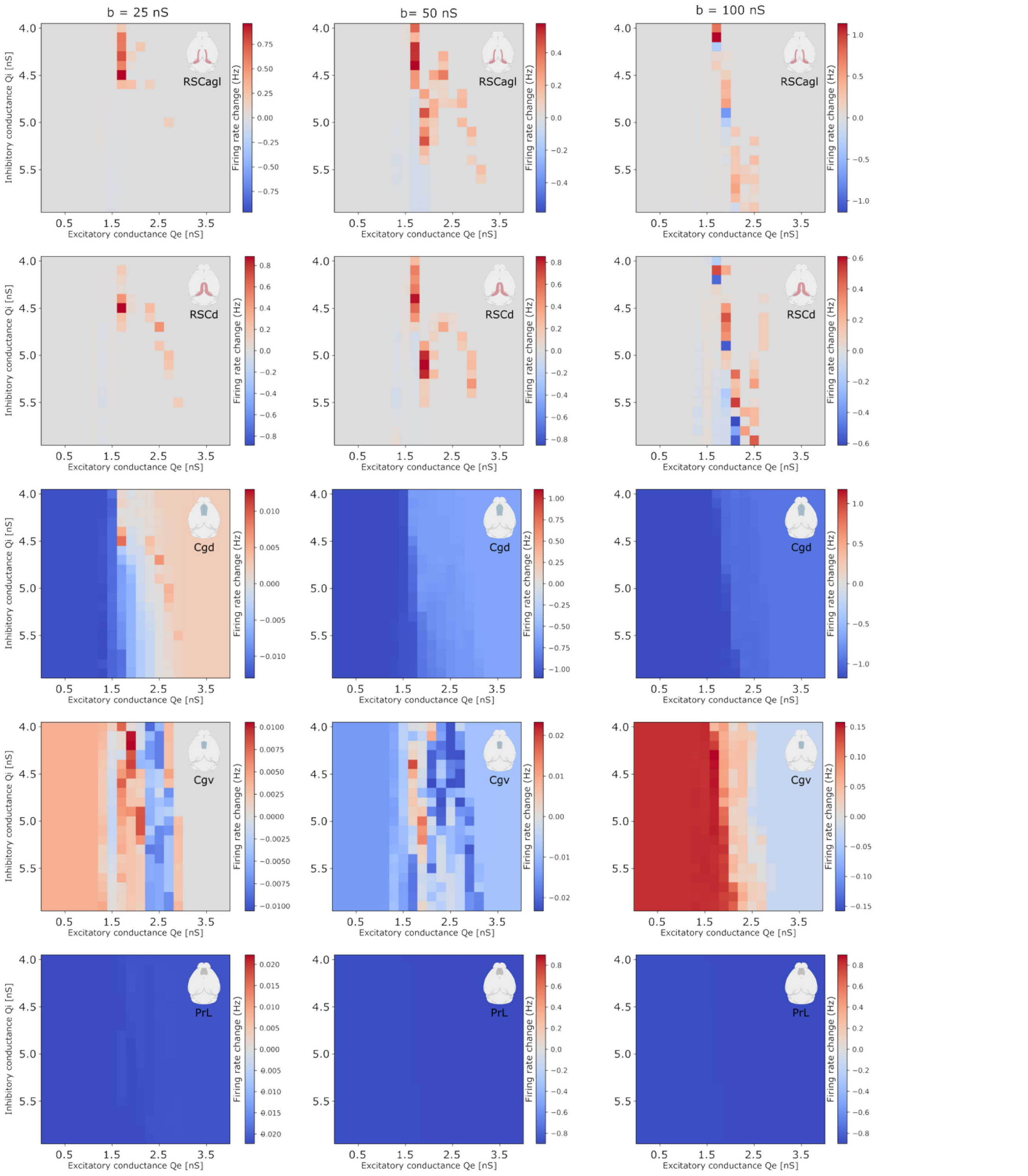

Figure S6. Effect of cholinergic neuromodulation via spike-frequency adaptation on DMN-wide suppression. Heatmaps of simulated responses in all DMN subregions except the RSCv, as a function of E and I synaptic conductances Q_E and Q_I in the RSC for varying levels of modeled cholinergic modulation via spike-frequency adaptation b and a fixed E-to-I/E-to-E ratio of 1.4.

bioRxiv preprint doi: https://doi.org/10.1101/2025.04.16.649208; this version posted February 16, 2026. The copyright holder for this preprint (which was not certified by peer review) is the author/funder, who has granted bioRxiv a license to display the preprint in perpetuity. It is made available under aCC-BY 4.0 International license.

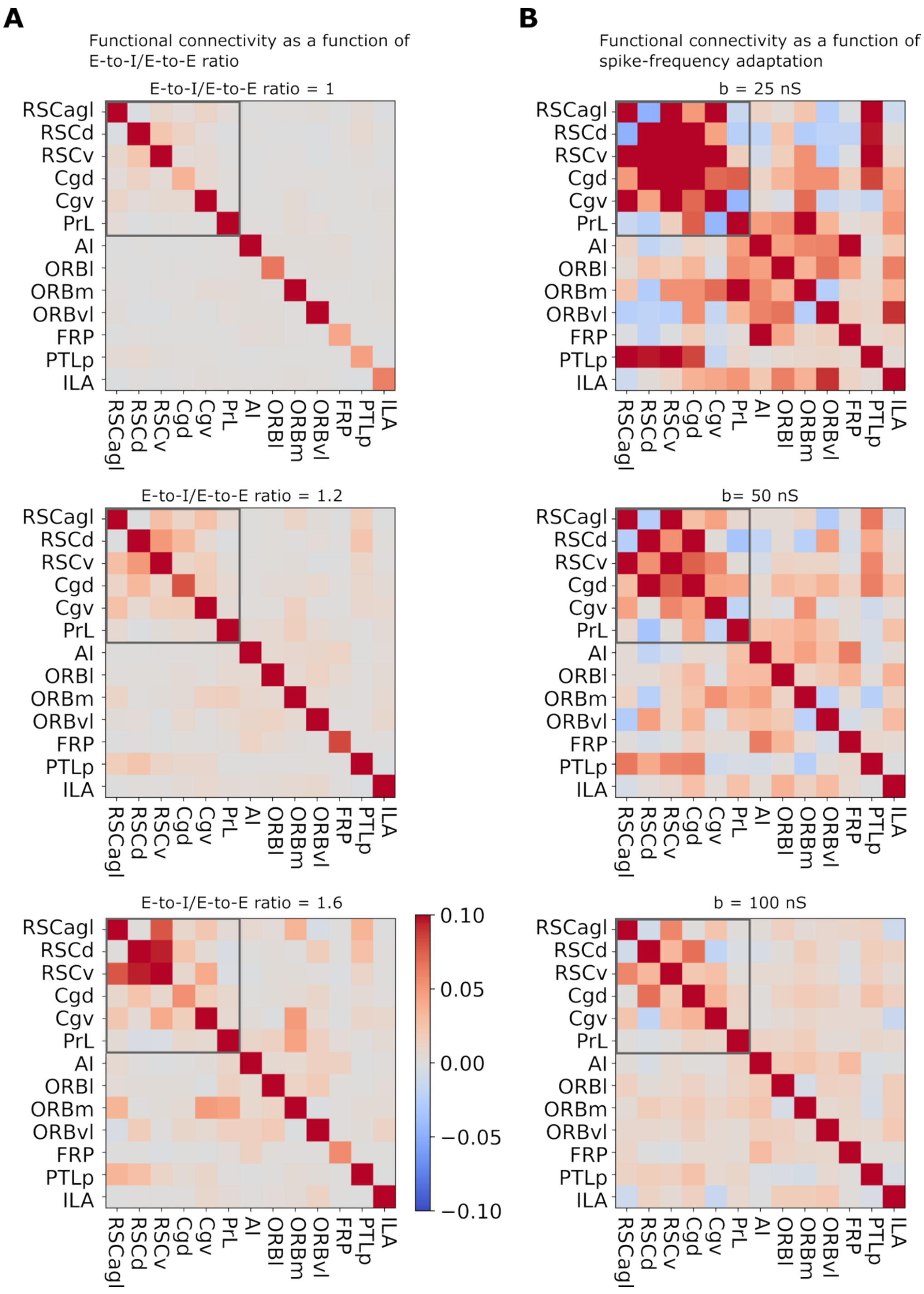

Figure S7. Resting-state functional connectivity matrices between the DMN, insula, and putative FN for different values of E-to-I/E-to-E ratio (**A**) and spike-frequency adaptation (**B**) model parameters. The results show intra-DMN functional connectivity growing with E-to-I/E-to-E ratio, as well as intra-DMN negative functional connectivity emerging in the presence of spike-frequency adaptation.

bioRxiv preprint doi: https://doi.org/10.1101/2025.04.16.649208; this version posted February 16, 2026. The copyright holder for this preprint (which was not certified by peer review) is the author/funder, who has granted bioRxiv a license to display the preprint in perpetuity. It is made available under aCC-BY 4.0 International license.

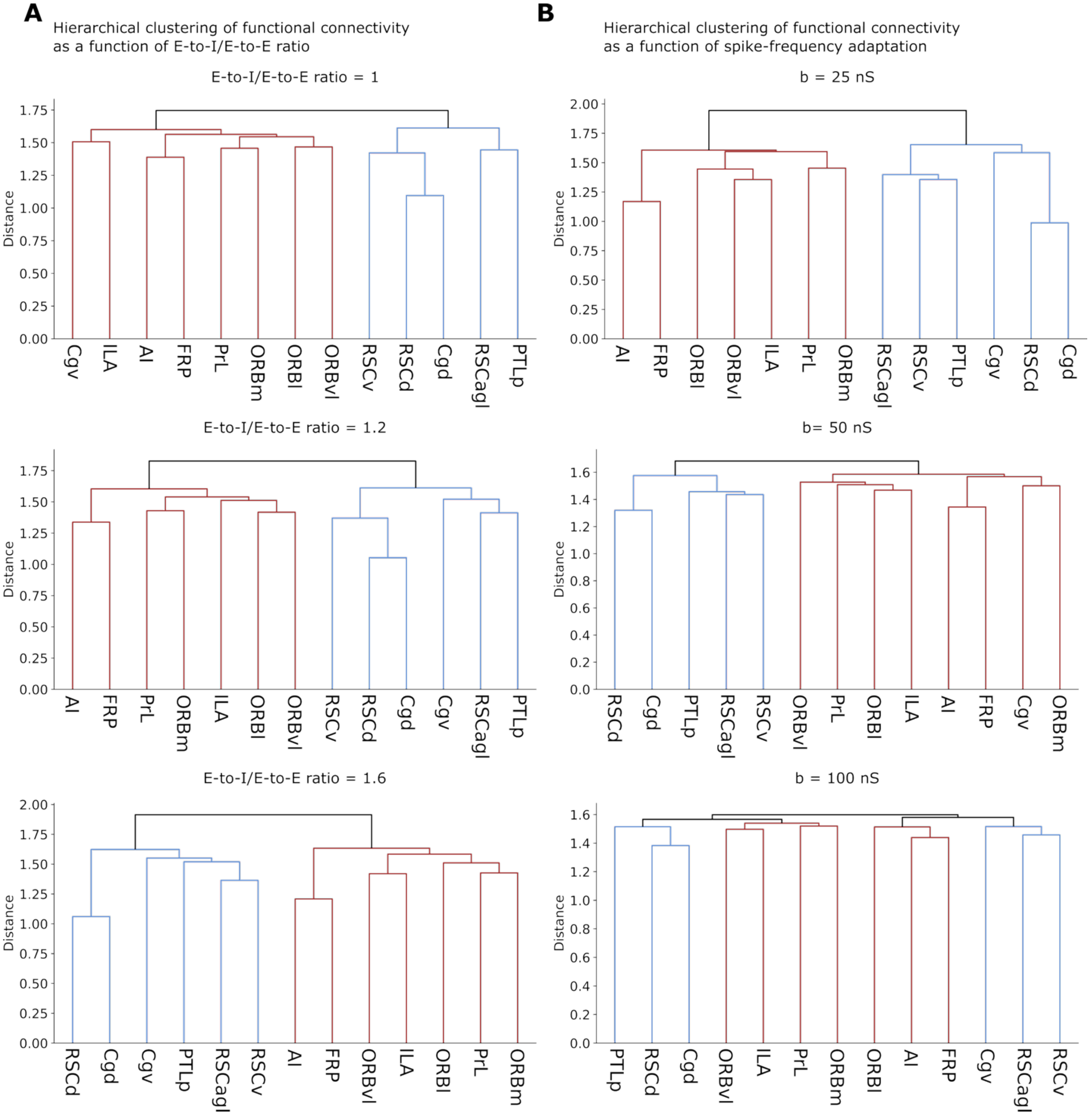

Figure S8. Resting-state functional connectivity hierarchical clustering dendrograms for different values of E-to-I/E-to-E ratio (**A**) and spike-frequency adaptation (**B**) model parameters. The results show the robustness of DMN emergence (in blue) across simulation parameters, as well as DMN breakdown at high spike-frequency adaptation.

bioRxiv preprint doi: https://doi.org/10.1101/2025.04.16.649208; this version posted February 16, 2026. The copyright holder for this preprint (which was not certified by peer review) is the author/funder, who has granted bioRxiv a license to display the preprint in perpetuity. It is made available under aCC-BY 4.0 International license.

**Feature**	**Parameter Range (local)**	**Parameter Range Global (EI to EE)**	**Parameter Range Adaptation**	**DMN Behavior**	**Interpretation**
**DMN suppression**	$Q_E = 1.3$ to 1.5 nS, $Q_I > 5$ nS, Or $Q_E = 1.5$, $Q_I > 4$ nS	Ratio > 1	$b < 100$ nS	Suppression in RSCv, Cgv, and PrL	Specific E and I conductance ranges are needed to simulate DMN suppression
**Loss of RSC response, DMN response fragmentation**	$Q_E = 1.4$ to 1.5 nS, $Q_I < 5$ nS	Ratio > 1	$b < 100$ nS	Suppression in Cgv and PrL, no response in RSCv	Specific E and I conductance ranges are needed to simulate DMN suppression
	$Q_E < 0.7$ or $Q_E > 1.7$ nS	Ratio >= 1	$b = 0$ nS		Specific E and I conductance ranges are needed to simulate DMN suppression
	$Q_E = 1.3$ to 1.5 nS, $Q_I > 5$ nS, Or $Q_E = 1.5$, $Q_I > 4$ nS	Ratio = 1	$b = 0$ nS		DMN suppression is robust to ratio provided ratio > 1
	$Q_E = 1.3$ to 1.5 nS, $Q_I > 5$ nS, Or $Q_E = 1.5$, $Q_I > 4$ nS	Ratio = 1.4	$b = 100$ nS	Suppression in PrL, no response in RSCv, enhancement in Cgv	High adaptation disrupts intra-DMN and DMN-insula interactions
**RSC reversal, DMN response fragmentation**	$Q_E = 0.7$ to 1.4 nS, $Q_I > 4$ nS	Ratio = 1.4	$b = 0$ nS	Suppression in PrL and Cg, enhancement in RSCv	Low activity due to low excitability, promoting enhancement over suppression by stimulation
	$Q_E = 1.6$ nS, $Q_I = 4.9$ to 5.5 nS	Ratio = 1.4	$b = 0$ nS	Suppression in PrL, enhancement in RSCv and Cgv	High excitability leads to distributed enhancement
	$Q_E > 1.5$ nS, $Q_I > 4$ nS	Ratio = 1	$b > 0$ nS	Suppression in PrL, enhancement in RSCv, no consistent response in Cgv	Adaptation reduces firing rate, promoting enhancement over suppression by stimulation

Table S1. Three distinct DMN breakdown modes. Our analysis revealed that simulated DMN suppression fails through three mechanistically distinct modes: loss of responsiveness, reversal from suppression to enhancement, and network fragmentation.

bioRxiv preprint doi: https://doi.org/10.1101/2025.04.16.649208; this version posted February 16, 2026. The copyright holder for this preprint (which was not certified by peer review) is the author/funder, who has granted bioRxiv a license to display the preprint in perpetuity. It is made available under aCC-BY 4.0 International license.

Feature	Parameter Range Global (EI to EE)	Parameter Range Adaptation	DMN Behavior	Interpretation
**DMN emergence as a network**	Ratio > 1	$b < 100\ nS$	DMN clusters as a network apart from insula and frontal network	DMN robustly emerges as a network across parameter regimes
**DMN reduced functional connectivity**	Ratio = 1	$b = 0\ nS$	DMN remains a separate cluster but with reduced internal functional connectivity	Sufficiently strong E-to-I connectivity maintaining global E-I balance is needed for strong intra-DMN connectivity
**DMN spontaneous fragmentation**	Ratio = 1.4	$b = 100\ nS$	DMN splits into two clusters: RSCv, RSCagl, and Cgv in one cluster, RSCd, Cgd, and PTLp in the other	Adaptation generates neural self-inhibition that gives rise to anticorrelation between DMN nodes

Table S2. Robustness of DMN emergence and parameter regimes for DMN integrity. DMN emergence as a distinct network is only disrupted at high spike-frequency adaptation rates. Although E-to-I/E-to-I inter-regional connectivity ratio influences functional connectivity in the DMN, it does not disrupt DMN integrity.
